# Structure and Binding Properties to Blood Co-Factors of the Least Sulfated Galactan Found in the Cell Wall of the Red Alga *Botryocladia occidentalis*

**DOI:** 10.3390/md22020081

**Published:** 2024-02-09

**Authors:** Antim K. Maurya, Hoda Al. Ahmed, Anderson DeWitt, Anter A. Shami, Sandeep K. Misra, Vitor H. Pomin

**Affiliations:** 1 Department of BioMolecular Sciences, University of Mississippi, Oxford, MS 38677, USA; akmaurya@olemiss.edu (A.K.M.);; 2 Research Institute of Pharmaceutical Sciences, School of Pharmacy, University of Mississippi, Oxford, MS 38677, USA

**Keywords:** blood co-factors, *Botryocladia occidentalis*, NMR, seaweed, SPR, sulfated galactan

## Abstract

Three different populations of sulfated polysaccharides can be found in the cell wall of the red alga *Botryocladia occidentalis*. In a previous work, the structures of the two more sulfated polysaccharides were revised. In this work, NMR-based structural analysis was performed on the least sulfated polysaccharide and its chemically modified derivatives. Results have revealed the presence of both 4-linked *α*- and 3-linked *β*-galactose units having the following chemical features: more than half of the total galactose units are not sulfated, the *α*-units occur primarily as 3,6-anhydrogalactose units either 2-*O*-methylated or 2-*O*-sulfated, and the *β*-galactose units can be 4-*O*-sulfated or 2,4-*O*-disulfated. SPR-based results indicated weaker binding of the least sulfated galactan to thrombin, factor Xa, and antithrombin, but stronger binding to heparin cofactor II than unfractionated heparin. This report together with our previous publication completes the structural characterization of the three polysaccharides found in the cell wall of the red alga *B. occidentalis* and correlates the impact of their composing chemical groups with the levels of interaction with the blood co-factors.

## 1. Introduction

Marine organisms represent an abundant source of medicinally relevant molecules [1]. Sulfated polysaccharides derived from marine algae can exhibit unique structures and specific biomedical properties with distinct mechanisms of action as compared to equivalent mammalian sulfated polysaccharides [2,3,4,5]. A common type of polysaccharide found in algae is sulfated galactan (SG) [6,7]. Algal SGs are found primarily in the cell walls of red and green seaweeds. Structurally speaking, these glycans naturally occur heavily sulfated, within polymers of high molecular weights (MWs), usually over 100 kDa, and composed mostly of both 3-linked *β*-D-galactose (Gal) and 4-linked *α*-L/D-Gal units. The D/L absolute configurations on the 4-linked *α*-Gal determine if the algal SG is classified as a carrageenan (D chirality), an agaran (L chirality), or a hybrid (both D/L chiralities) [7]. Other modifications such as the occurrence of 3,6-anhydrogalactose (AnGal) units in the backbone and hydroxyl substitutions by methylation, acetylation, and pyruvylation can be also found in algal SGs [6,8,9,10,11,12]. Although all these modifications add up to the overall structural complexity in this class of polysaccharide, this structural complexity makes the chemical and functional research of these molecules limitless and favorable, providing, consequently, a vast repertoire of potentially new medicinal glycans of unique structures and distinct mechanisms of action [6,13,14]. Perhaps the most studied, and surely some of the first researched, potential biomedical activities of the algal SGs are those related to their inhibitory properties of clotting and thrombus formation [15,16,17]. Qualitative analysis of molecular binding of the algal SGs with target blood co-factors is, therefore, essential in drug discovery and the development of these marine compounds.

*Botryocladia occidentalis*, a red alga from the Rhodymeniacea family, has been taking a special place among the many seaweeds studied regarding the structure and biological activities of SGs. One of the first publications regarding the structure and potential anticoagulant action of *B. occidentalis*-derived SGs (BoSGs) dates to more than two decades ago [17]. In this work, three populations of sulfated polysaccharides were isolated from the *B. occidentalis* cell wall by means of anion-exchange chromatography. D chirality was assigned in this reference to the 4-linked α-Gal units, classifying the BoSG in the group of carrageenans. Our recent publication was able to revise the structure of the two more sulfated BoSG fractions [12]. Numerous publications regarding the potential biomedical applications of these BoSG fractions have been documented in the literature [2,7,13,14,16,18,19,20,21,22,23,24,25,26,27,28,29,30]. But curiously, most of these publications have relied in their findings on the incorrect structure proposed in the original work [17], but not on the revised structure [12]. In addition, none of these works published have investigated the potential biomedical use and/or revisited the structure of the least sulfated BoSG fraction.

In this current work, after proper isolation of the three main BoSG populations found in the cell wall of *B. occidentalis*, multiple physicochemical analyses were conducted on the least sulfated BoSG fraction. For this, various techniques were performed, including one-dimensional (1D) and two-dimensional (2D) nuclear magnetic resonance (NMR) spectroscopy, high performance size-exclusion chromatography/multi-angle light scattering (HPSEC/MALS), polyacrylamide gel electrophoresis (PAGE), and surface plasmon resonance (SPR) spectroscopy. The 2D NMR methods used were ^1^H/^1^H correlation spectroscopy (COSY), ^1^H/^1^H total correlation spectroscopy (TOCSY), and ^1^H/^13^C-edited heteronuclear single quantum coherence (HSQC) spectra. Chemically modified derivatives, such as desulfated, oversulfated, and alkali-treated (AnGal-enriched), were also employed in our study to aid our understanding of the structural and functional properties of this less studied BoSG fraction. SPR analyses were conducted using a heparin surface in a competitive system against the four main binding blood co-factors: thrombin (IIa), factor Xa, antithrombin (AT), and heparin cofactor II (HCII). This work, together with our previous publication [12], concludes the structural characterization of all three BoSGs and offers a correlation between their respective composing structural features and the quality of their interactions with the key binding coagulation co-factors.

## 2. Results and Discussion

### 2.1. Isolation and Preliminary Physicochemical Analyses of BoSG Fr1

**Figure 1:**
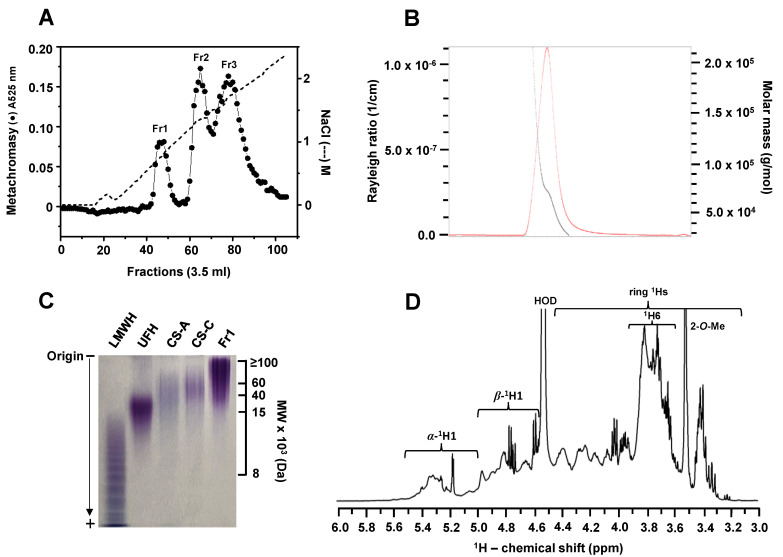
(**A**) Fractionation, (**B**,**C**) MW analyses, and (**C**) 1D ^1^H NMR spectrum of the *B. occidentalis*-derived sulfated galactan fraction 1 (BoSG Fr1). (**A**) Purification of BoSG Fr1 was conducted by DEAE Sephacel anion-exchange chromatography. The column was eluted through a linear gradient of 0–3.0 M NaCl in 50 mM sodium acetate buffer (pH 6.0). The flow rate was set at 3.5 mL/10 min, and after collection, fractions were assayed for their metachromatic properties at 525 nm (-●-). The NaCl concentration was checked by conductivity (---). Three eluent fractions were identified and collected: Fr1, Fr2, and Fr3. (**B**) HPSEC-MALS profile, and (**C**) PAGE analysis of BoSG Fr1. The rough MWs of the reference compounds used in PAGE (LMWH, UFH, CS-A, and CS-C) are indicated at the right of the gel. The samples were loaded at the top of the gel and migrated towards the anode at the bottom of the gel. The migration direction is indicated by the arrow on the left of the gel. (**D**) 1D ^1^H NMR spectrum of BoSG Fr1 (δ_H_ expansion from 6.0 to 3.0 ppm). About 5.0 mg of BoSG Fr1 was dissolved in 0.5 mL D_2_O and the 1D ^1^H NMR spectrum was recorded at 50 °C through a 500 MHz Bruker NMR instrument. The spectral regions of the anomeric ^1^H signals of the *α*- and *β*-units, the ^1^H6, the 2-methoxy, and ring protons are indicated in the spectrum.

The mixture of the major sulfated polysaccharides, namely BoSGs, from the red alga *B. occidentalis* was obtained through a papainolytic digestion of its dried cell wall. Three distinct BoSG populations of different polyanionic characters were purified from this extract via anion-exchange chromatography on a DEAE Sephacel column eluted with a linear 1–3 M NaCl gradient (Figure 1A). The polysaccharide fractions were monitored post-column by metachromasy (absorbance at 525 nm) using the dye 1,9-dimethylmethylene blue (DMB). The resultant chromatogram of this assay (Figure 1A) depicts three main peaks: the first one, labeled as Fr1, presumably with the lowest sulfation content, and two other peaks with gradually higher amounts of sulfation, labeled as Fr2 and Fr3, respectively [12]. These peaks were eluted from the column at NaCl concentrations of 0.5, 1.2, and 1.5 M, respectively, as indicated by the dotted line in Figure 1A. The first peak (BoSG Fr1), unstudied in previous reports of BoSGs, was subjected here to a series of analytical techniques for assessment of MW, polydispersity, full structural characterization, and binding properties to blood co-factors.

Results from the HPSEC/MALS analysis (Figure 1B) indicate that BoSG Fr1 has a MW of 70.24 ± 2.144 kDa. Electrophoretic migration of BoSG Fr1 was assessed by PAGE to monitor molecular dispersity in comparison to other reference sulfated glycans such as low molecular weight heparin (LMWH), unfractionated heparin (UFH), chondroitin sulfate A (CS-A), and chondroitin sulfate C (CS-C). The PAGE results clearly indicated a polydispersed material of BoSG Fr1 composed of a mixture of multiple chains whose MWs fall roughly within the range of 20 to ≥100 kDa (Figure 1C). The HPSEC/MALS and PAGE data corroborate well in the MW assessment of the BoSG Fr1. The 1D ^1^H NMR spectrum of this fraction (Figure 1D) showed NMR signal profiles characteristic of SGs composed of two sets of anomeric (^1^H1) signals: one belonging to the *α*-Gal units resonating at the most downfield ^1^H1 region of the spectrum (δ_H_ between 5.6 and 5.0 ppm), and one belonging to the *β*-Gal residues resonating at the most upfield ^1^H1 region of the spectrum (δ_H_ between 5.0 and 4.6 ppm). Other ^1^H signals were also identified via NMR such as the ring ^1^Hs (δ_H_ between 4.5 and 3.2 ppm), and those belonging to the chemical groups outside the hexose ring such as CH_2_ (δ_H_ between 3.9 and 3.6 ppm), and CH_3_ (δ_H_ around 3.5 ppm), as discussed further, based on the 2D NMR analyses.

### 2.2. Structural Characterization of BoSG Fr1 by NMR Spectroscopy

**Figure 2:**
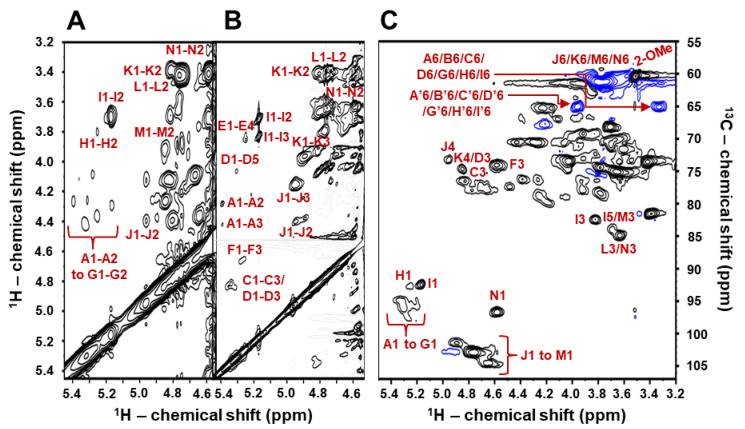
(**A**) 2D ^1^H/^1^H COSY and (**B**) ^1^H/^1^H TOCSY, and (**C**) ^1^H/^13^C-edited HSQC NMR spectra of native BoSG Fr1. Strip of the anomeric region (δ_H_/δ_H_ expansions 5.45–4.54/3.2–5.45 ppm) of (**A**) COSY, and (**B**) TOCSY spectra indicating, respectively, the short-range ^1^H/^1^H connectivities and long-range ^1^H/^1^H spin systems. (**C**) ^1^H/^13^C HSQC spectrum (δ_H_/δ_C_ expansions 5.5–3.2/55.0–107.0 ppm) showing the ^1^H/^13^C cross-peaks of the composing sugar rings. All 2D NMR spectra were recorded in D_2_O at 50 °C on a 500 MHz Bruker NMR instrument. The peaks with blue contours represent the negative phase of the CH_2_ groups, whereas the peaks with black contours represent the positive phase of the CH and CH_3_ groups.

Structural determination of the composing Gal units in BoSG Fr1 was achieved by a combination of multiple 2D NMR spectra including ^1^H/^1^H COSY for assessment of short-range carbon-bonded vicinal/geminal ^1^Hs (Figure 2A), ^1^H/^1^H TOCSY for assessment of long-range carbon-bonded ^1^H spin systems (Figure 2B), and ^1^H/^13^C-edited HSQC for assessment of proton–carbon pairs (Figure 2C). For illustrative purposes, only the anomeric ^1^H region of the COSY and TOCSY spectra are depicted in Figure 2A,B. After proper assignments of all ^1^H chemical shifts (δ_H_) of all Gal units using COSY and TOCSY spectra, the ^13^C chemical shifts (δ_C_) were assigned using the HSQC spectrum, and full ^1^H and ^13^C chemical shift assignments were obtained for all Gal units of the native BoSG Fr1 (Table 1). A system of letters (from A to N) followed by numbers (from 1 to 6) was used to assign and label, in the 2D NMR spectra, all the δ_H_ and δ_C_ within the hexose rings of the distinct Gal units in BoSG Fr1 (Figure 2, and Table 1). Comparison between the δ_H_ and δ_C_ values from BoSG Fr1 with reference values from the literature [31,32,33,34] (Table 1), and the typical downfield δ_H_ shift of ~0.6 ppm, and downfield δ_C_ shift of ~6 ppm of sulfation (bold values) and glycosylation (italic values) sites have enabled us to identify the sulfation patterns and glycosidic bonds in the Gal units of BoSG Fr1. Typical δ_H_ and δ_C_ values of other ring positions have assisted us in identifying the presence of other chemical modifications such as the 3,6-anhydro Gal moiety in the *α*-AnGal units as well as the presence of methylation.

The sulfation patterns of the *α*-AnGal units labeled as A, C, F, and G were determined by the downfield δ_H_/δ_C_ seen at ring position 2 of their units (δ_H_/δ_C_ at 4.27/78.7, 4.41/76.1, 4.36/76.1, and 4.29/78.5 ppm, respectively) (Table 1). These shifts distinctly revealed the presence of 2-*O*-sulfation on the *α*-AnGal residues. The characteristic δ_H_/δ_C_ pair of 3.51/60.1 ppm additionally observed in all 2D NMR spectra, besides those of the ring atoms (^1^H1/^1^H6 and ^13^C1/^13^C6), were diagnostic of the methoxy group (*O*-CH_3_) attached to the ring position 2 in *α*-AnGal residues labeled as B and E (Table 1). The *α*-AnGal units labeled as B, D, E, H, and I were assigned as non-sulfated units, as seen from the lack of downfield chemical shifts in their ring ^1^H and ^13^C nuclei (Table 1). The typical downfield resonances of δ_H_/δ_C_ at ~4.4/76 ppm and δ_H_/δ_C_ at ~4.9/74 ppm in the *β*-Gal units, residues labeled as J and K, are diagnostic of 2-*O*- and 4-*O*-sulfation, respectively (Table 1). Note that only the *β*-Gal unit labeled as J bears these downfield resonances, indicating, therefore, 2,4-*O*-di-sulfation at this unit, while the *β*-Gal unit labeled as K shows only the downfield resonance at the ring position 4, indicating, therefore, a 4-*O*-sulfated unit. The *β*-Gal units labeled as L, M, and N were assigned as non-sulfated units, as seen from the lack of downfield chemical shifts in their ring ^1^H and ^13^C nuclei (Table 1). The relative integrals of the NMR signals related to sulfated:non-sulfated Gal units in BoSG Fr1 were calculated as 40:60%.

**Figure 3:**
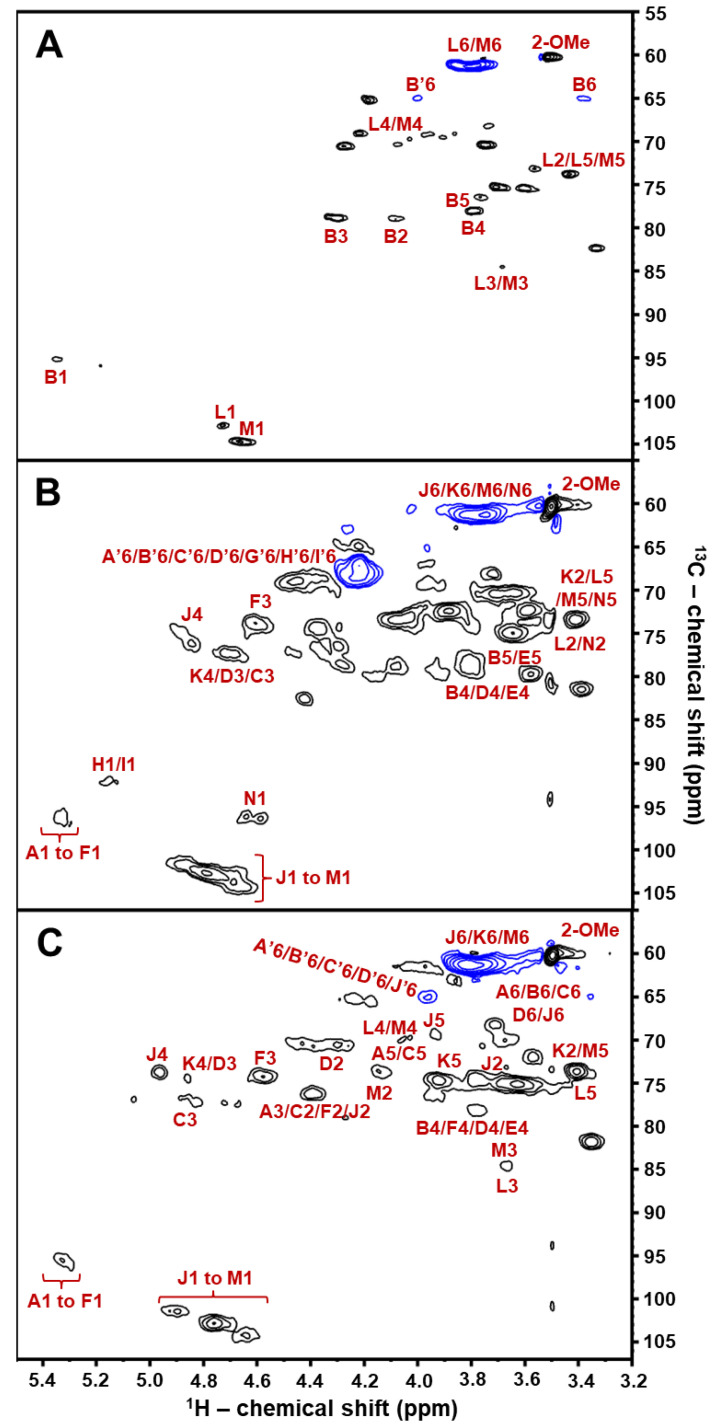
2D ^1^H/^13^C HSQC NMR spectra (δ_H_/δ_C_ expansions 5.5–3.2/107.0–55.0 ppm) of the three chemically modified BoSG Fr1 derivatives studied in this work: (**A**) desulfated, (**B**) oversulfated, and (**C**) alkali-treated AnGal-enriched derivatives with key ^1^H/^13^C cross-peaks properly assigned. All 2D NMR spectra were recorded in D_2_O at 50 °C on a 500 MHz Bruker NMR instrument. The peaks with blue contours represent the negative phase of the CH_2_ groups, whereas the peaks with black contours represent the positive phase of the CH and CH_3_ groups.

**Figure 4:**
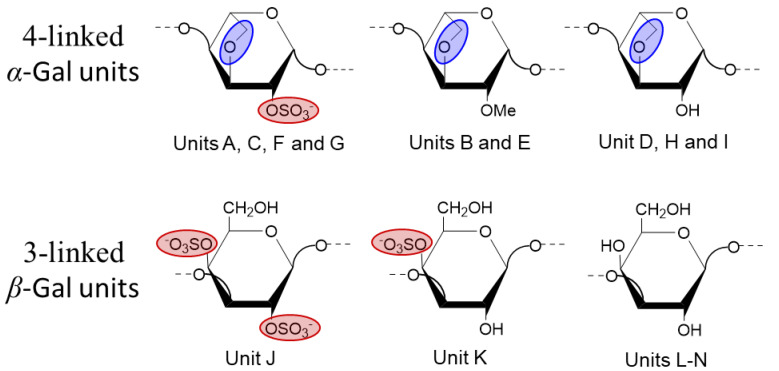
Structural representation of the BoSG Fr1 units identified by NMR analyses. The backbone of BoSG Fr1 is composed of both 4-linked *α*-Gal and 3-linked *β*-Gal units along with a variety of chemical modifications including sulfation at different levels and positions, presence of 3,6-anhydro moiety (AnGal), and methylation at the 2-*O* position. Sulfation esters are presented by red ellipses, while the 3,6-anhydro moiety is highlighted with blue ellipses. The letters used in the NMR assignments are indicated in the structures.

Chemical derivatives of BoSG Fr1, particularly desulfated, oversulfated, and alkali-treated (AnGal-enriched) samples, were synthesized to confirm our ^1^H/^13^C NMR spectral assignments of the sulfation patterns, and the 3,6-anhydro moiety in the Gal units of BoSG Fr1. ^1^H/^13^C HSQC spectra recorded on these derivatives are displayed in Figure 3. As expected with the desulfation process, most of the positive-phased ^1^H/^13^C resonances related to sulfated sites with a δ_H_/δ_C_ range of 5.0–4.4/80–70 ppm disappeared in the HSQC spectrum of the desulfated derivative (Figure 3A), together with the intensity enhancement of the negative-phased resonances assigned as L6/M6 with δ_H_/δ_C_ at ~3.7/60 ppm and the decrease in the sulfate-related ^1^H6/^13^C6 cross-peaks with δ_C_ at 65 ppm (Figure 2C). As expected, the HSQC spectrum of the oversulfated BoSG Fr1 showed enhancement in signal intensity of all sulfated related ^1^H/^13^C cross-peaks such as the negative-phased, blue-contoured peak with δ_H_/δ_C_ around 4.25/67 ppm, and the black-contoured peaks with a δ_H_/δ_C_ range of 5.0–4.4/80–70 ppm (Figure 3B). The presence of the *α*-AnGal units in BoSG Fr1 was affirmed by the ^1^H/^13^C HSQC spectral analysis of the alkali-treated derivative (Figure 3C) as compared to the HSQC spectrum of the native BoSG Fr1 (Figure 2C). The levels of signal intensity observed for the CH_2_-related negative-phased ^1^H/^13^C cross-peaks in both spectra were very similar, indicating that the *α*-Gal units in BoSG Fr1 occur primarily in the form of 3,6-anhydro units, AnGal. In all, the ^1^H/^13^C assignments made in the three chemical derivatives (Figure 3) were very useful, either to assist with or to confirm some of the ^1^H/^13^C assignments made on the native BoSG Fr1 (Table 1). Figure 4 depicts the structures of all Gal units characterized in BoSG Fr1 by NMR spectroscopy.

### 2.3. SPR-Based Binding Properties of BoSG Fr1 and Derivatives

**Figure 5:**
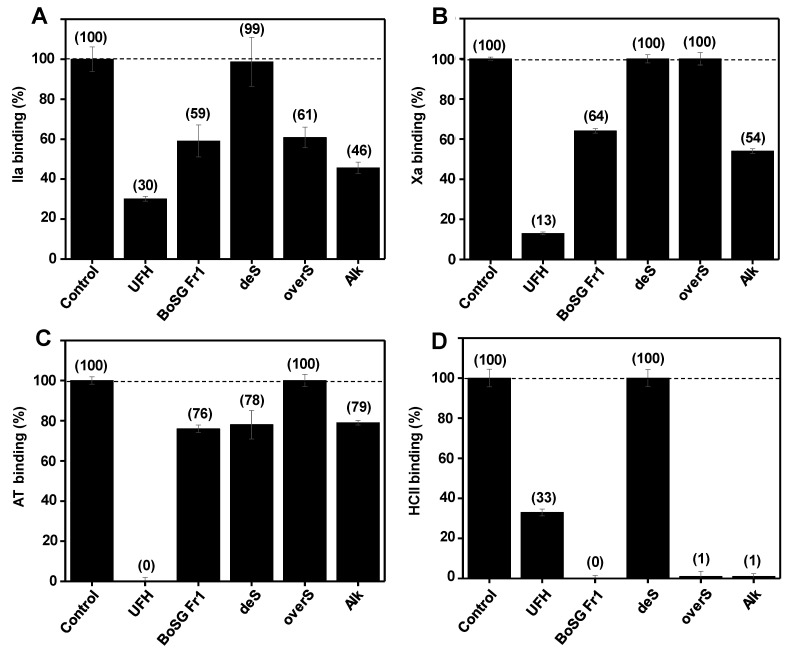
Solution SPR competitive effect of native BoSG Fr1 and its derivatives on binding of blood (co)-factors to heparin surface. Bar graphs (based on triplicate experiments with standard deviation) of normalized (**A**) IIa, (**B**) Xa, (**C**) AT, and (**D**) HCII binding to heparin surface in absence of any glycan (negative control), or presence of UFH (positive control), BoSG Fr1, and its three chemical derivatives: desulfated (deS), oversulfated (overS), and alkali-treated (Alk). The numbers on top of each bar indicate the average normalized binding value obtained in the experiments.

Solution SPR-based analyses were performed in a competitive fashion using a heparin surface (sensor chip) as previously reported [12]. In this study, the quality of interactions between the four BoSG Fr-1-derived samples used in our NMR-based analyses, native, desulfated (deS), oversulfated (overS), and alkali-treated (Alk) and the four main binding blood co-factors, IIa, Xa, AT, and HCII, were measured in terms of the binding inhibitory percentage of each co-factor alone (Figure 5). The standard mammalian glycosaminoglycan of high anticoagulant activity, UFH, was used in each assay as a positive control. Native BoSG Fr1 showed weaker binding inhibition of IIa (40% BoSG Fr1 vs. 70% UFH) (Figure 5A), Xa (35% BoSG Fr1 vs. 85% UFH) (Figure 5B), and AT (25% BoSG Fr1 vs. 100% UFH) (Figure 5C) than UFH, but stronger binding inhibition of HCII than UFH (100% BoSG Fr1 vs. 65% UFH) (Figure 5D). Taking into consideration a comparative analysis of the binding inhibitory property of BoSG Fr1 to the four blood co-factors against the heparin sensor chip, the following order of binding preference could be observed: HCII > IIa = Xa >> AT. This observation proves the preference of BoSG Fr1 for HCII and the weaker binding property of UFH to HCII as compared to BoSG Fr1. The necessity to use a higher concentration of HCII (800 nM) for interaction with the heparin surface during the SPR assay corroborates the fact that HCII does not bind to the heparin (sensor chip) as strongly as the other co-factors.

As expected, the binding inhibition of BoSG Fr1 was compromised by desulfation, except for the case against AT, in which the weak inhibition did not change from the native molecule. Interestingly, oversulfation did not improve the binding inhibition, meaning that the interaction of the native BoSG Fr1 to all four coagulation co-factors cannot be enhanced by oversulfation. The similar binding inhibition of the alkali-treated sample as compared to the native sample indicates that this reaction does not influence the binding property of BoSG Fr1 to the four blood co-factors. This SPR-based observation agrees with our NMR-based study that the native BoSG Fr1 is already fully composed of AnGal units, and the alkali treatment of the original sample does not significantly change its structure. Overall, BoSG F1 displays a relatively weak binding property to the four blood co-factors, and this might be a result of the structure of this BoSG fraction, composed of a low sulfation content (40% of all Gal units) and the presence in significant amounts of the AnGal units and *O*-methylation. The occurrence of AnGal units was already proved in our previous work [12] to be deleterious in binding to the coagulation proteases (IIa and Xa), and serpins (AT and HCII), as well as to the catalytic inhibition of these serpins over these proteases in the presence of the exogenous polysaccharide [12]. Methylation is generally accepted to result in a chemical group that does not enhance the interacting property of sulfated polysaccharides to binding proteins, especially when electrostatic interactions result from the negatively charged sulfate groups in sulfated polysaccharides, and the positively charged amino acid patches in the protein partners are, overall, governing the binding quality.

## 3. Materials and Methods

### 3.1. Reagents

Papain (# P4762), EDTA (# 03609), cysteine (# C7352), cetylpyridinium chloride (CPC) (# C0732), sephadex G-15 resin (# G15120), DMB, and UFH (180 IU/mg) were purchased from Sigma-Aldrich (Saint Louis, MO, USA). Dialysis membrane (# 9201735, Spectra/Por) was procured from Spectrum Laboratories, Inc. (Rancho, Dominguez, CA, USA). Sodium acetate (# S22040) was purchased from RPI, Mt. Prospect, IL, USA. DEAE Sephacel resin (# 17-0500-01) was obtained from Cytiva, Marlborough, MA, USA. The polystyrene chromatography columns (2.5 × 20 cm, # 7371598, and 1.0 × 30 cm, # 7376607) used for purification and desalting of purified compound were procured from Bio-Rad Laboratories, Hercules, CA, USA. The coagulation factors Xa, IIa, AT, and HCII were purchased from Haematologic Technologies (Huissen, The Netherlands). Amine-PEG3-Biotin was from Pierce, Rockford, IL, USA. HEPES [4-(2-hydroxyethyl)-1-piperazineethanesulfonic acid] was from Fisher Scientific. Biotin-Streptavidin (SA) Sensor Kit was purchased from Nicoya (Kitchener, ON, Canada).

### 3.2. Extraction of BoSGs

The marine red alga *B. occidentalis* was sourced from Gulf Coast Ecosystems (Ellenton, FL, USA). The BoSGs were extracted using the procedure described in a previous reference [12], with minor changes. The *B. occidentalis* cell wall was chopped into tiny pieces and then freeze-dried and lyophilized for 24 h. The resulting dried sample (2.5 g) was then treated for 24 h at 60 °C with papain (1 mg papain/g dry tissue), 5 mM EDTA, and 5 mM cysteine after being dissolved in a 0.1 M NaOAc buffer (pH 6.0). After this, the incubation mixture was centrifuged for 30 min at 3000 rpm to collect the supernatant. Then, crude polysaccharide solution was precipitated by adding 3 mL of 10% CPC solution and allowed to stand at room temperature for 24 h. The precipitate obtained was subjected to centrifugation for 30 min at 3000 rpm, then washed with 300 mL of a 5% CPC solution. After being centrifuged again, it was then dissolved in 86 mL of a solution containing 2 M NaCl and 15% ethanol (*v/v*). Subsequently, it was re-precipitated by the addition of 150 mL of absolute ethanol and allowed to incubate at −20 °C for 24 h. The polysaccharides were recovered through centrifugation with the same parameters as described above and washed two times with 150 mL of 80% ethanol followed by another wash with 150 mL of absolute ethanol. The resulting precipitate was subjected to lyophilization for 24 h to obtain crude polysaccharide. The crude polysaccharide powders were stored in a glass vial at 4 °C for further utilization.

### 3.3. Fractionation of BoSGs

The crude BoSG (50 mg) was firstly dissolved in 1 mL NaOAc (50 mM) buffer and subjected to purification with a DEAE Sephacel anion-exchange resin column (2.5 × 20 cm). Subsequently, the isolation procedure was achieved by gradually increasing the NaCl (in 50 mM NaOAc, pH 6.0) from 0 to 3 M at a flow rate of 21 mL/h. The specific BoSG Fr1 fractions (3.5 mL/tube) were then collected in 6 mL culture tubes. The eluted sugar fractions were determined using the DMB-based colorimetric assay (Figure 1A). Absorbance was recorded at 525 nm on a micro plate reader (SpectraMax ABS) from Molecular Devices, LLC (San Jose, CA, USA). The relevant fractions were combined, dialyzed against distilled water, freeze-dried, and lyophilized. To increase the quantity of the targeted compound, the procedure was repeated eight times. The total output was approximately 20 mg for Fr1. Conductivity experiments were applied to determine the NaCl concentration.

### 3.4. Desalting of BoSG Fr1

Every 10 mg of BoSG Fr1 obtained from the DEAE column was subjected to Sephadex G-15 in a column (1 × 25 cm) and eluted with distilled water (0.5 mL/min), and volumes of 1 mL/culture tube were collected. The metachromasy (DMB-based) method was used to determine sugar content, while precipitation with AgNO_3_ was used to determine NaCl content. The desalted BoSG Fr1 was obtained by pooling and lyophilization. Although Gal was already reported as the main monosaccharide constituent in BoSG, another round of monosaccharide analysis was performed (See Figure S1), proving unequivocally this sole composition.

### 3.5. Desulfation of BoSG Fr1

Chemical desulfation of BoSG Fr1 was performed according to previously published method [12,35,36]. Briefly, 5 mg of BoSG Fr1 was solubilized in 0.5 mL distilled water and then loaded to a Dowex 50-W (H^+^ 200–400 mesh) column. After the Dowex 50-W column, fractions containing sugar content were combined, neutralized with pyridine, and then submitted to lyophilization. Obtained pyridinium salt was dissolved in 0.5 mL DMSO/methanol (9:1 *v*/*v*) and heated at 80 °C for 6 h. The resulting desulfated product underwent dialysis against distilled water and was subsequently lyophilized, yielding 3.0 mg.

### 3.6. Oversulfation of BoSG Fr1

The BoSG Fr1 was oversulfated as described previously [12,37,38] with minor changes. Briefly, BoSG Fr1 (5.0 mg) was dissolved in distilled water (0.5 mL) and passed through a Dowex 50-W (H^+^ 200–400 mesh) column. Fractions, identified through metachromasy (DMB), were pooled, and a solution of 10% tributylamine in ethanol was added. Subsequently, the sample was then dialyzed and lyophilized. The obtained salt was dissolved in *N*,*N*-dimethylformamide (0.5 mL), and pyridine–sulfur trioxide (4 mol/equiv. of available hydroxyl group) was added. The reaction mixture was stirred for 1 h at 80 °C and quenched with the addition of water (1.6 mL). The resulting solution was diluted with three volumes of ethanol saturated with anhydrous sodium acetate and incubated at −20 °C for 1 h to formation of a white precipitate. After centrifugation at 3000 rpm × 30 min, the precipitate was collected. The oversulfated BoSG Fr1 precipitate was subsequently dissolved in water, subjected to dialysis, and finally lyophilized.

### 3.7. Alkaline Treatment

Alkaline treatment of BoSG Fr1 was conducted following previously reported protocols [12,39,40]. Briefly, 5.0 mg of BoSG Fr1 was dissolved in a 5% NaOH solution, and the resulting mixture was subjected to heating at 80 °C for 3 h. The alkali-treated product underwent dialysis against distilled water and was subsequently lyophilized. The final yield was determined to be 2.8 mg.

### 3.8. PAGE and HPSEC/MALS

The electrophoretic mobility of purified BoSG Fr1 was determined by PAGE along with known MWs including LMWH (~8 kDa), UFH (~15 kDa), CS-A (~40 kDa), and CS-C (~60 kDa) as published previously [12,41,42]. Briefly, a 1 mm thick discontinuous PAGE system having 4% stacking gel and a 12% resolving gel phases was prepared. A comb (8-tooth, well forming) was then equipped to create wells. For electrophoresis, the comb was removed, and 10 µg of each sample was subjected to electrophoresis at 100 V. Tris buffer (0.5 M, pH 6.8) was used as running electrophoresis. The movement of bands was monitored by 0.02% bromocresol green dye incorporated to the same gel. After electrophoresis, the gel was subjected to staining with 0.1% (*w*/*v*) toluidine blue dissolved in 1% acetic acid. Subsequently, destaining of gel was achieved with several washings with 1% acetic acid.

HPSEC was carried out utilizing an Ultimate 3000 high performance liquid chromatography system (Thermo Scientific, Sunnyvale, CA, USA) with an SEC column (Acquity BEH SEC column, 200 Å, 1.7 µm, 4.6 mm × 300 mm, Waters, Milford, MA, USA) to estimate the average MWs of BoSG Fr1. The eluent was monitored using MALS with a DAWN HELEOS II MALS detector (Wyatt Technologies Co., Santa Barbara, CA, USA). The instrument was calibrated with polystyrene in toluene for the MALS detector and bovine serum albumin for the MALS refractive index detectors. The sample was eluted isocratically on the SEC column with 50 mM ammonium acetate at a flow rate of 0.2 mL/min. To validate the measurement, MW 100 kDa dextran from Sigma-Aldrich Corp. (St. Louis, MO, USA) was utilized.

### 3.9. NMR Spectroscopy

About 5 mg of BoSG Fr1 was deuterium-exchanged three times through lyophilization using deuterium oxide (D_2_O, 99.9%; Sigma, Saint Louis, MO, USA). The resulting product was subsequently dissolved in 500 μL D_2_O and transferred to an NMR tube (5 mm diameter). All NMR experiments were carried out at 50 °C on 500 MHz Bruker Avance III HD equipped with 5 mm prodigy H/F-BBO cryoprobe. A set of NMR spectra was obtained, including 1D ^1^H, and 2D ^1^H/^13^C HSQC, ^1^H/^1^H COSY and ^1^H/^1^H TOCSY. 1D ^1^H NMR spectra were collected using the following parameters: relaxation delay of 1.0 s, acquisition time of 3.2 s, and number of scans of 128. ^1^H/^13^C HSQC spectrum of purified compounds was acquired using the following additional settings: acquisition times T_1_ of 0.146 s and T_2_ of 0.003 s, using FID data points of 1024 × 128. HSQC acquisition was executed employing a double INEPT transfer method, incorporating Echo/Antiecho TPPI gradient selection with decoupling during acquisition and using trim pulses in INEPT transfer. ^1^H/^1^H COSY and ^1^H/^1^H TOCSY spectra were obtained using T_1_ of 0.5 s and T_2_ of 0.03 s. The acquisition parameters included a total of 256 scans, with a spin-lock duration of 0.08 s specifically for the TOCSY spectrum. The obtained free induction decay file of TOCSY was processed by zero filling and linear prediction prior to Fourier transform. In all 2D NMR experiments, delays of five times T_1_ relaxation times were included between multiple pulses to ensure full recovery of magnetization during the experimentation. MestReNova 14.1.0 and TopSpin 3.6.4 software (Bruker, Ettlingen, Germany) were used to process and analyze the acquired NMR spectra. The identified structures were designed using ChemDraw 21.0.0 software.

### 3.10. SPR Spectroscopy

The biotinylated heparin was prepared using the following method: 2 mg of UFH and 2 mg of amine-PEG3-Biotin were dissolved in 200 μL H_2_O, then 10 mg of NaCNBH_3_ was added. The reaction mixture was heated in an oil bath at 70 °C for 24 h with continuous stirring. After 24 h, another 10 mg of NaCNBH_3_ was added, and the reaction was heated at 70 °C for another 24 h. The mixture was cooled to room temperature and desalted with a centrifugal filter (3000 MWCO). Biotinylated heparin was collected from the filter top, freeze-dried, and used for immobilization on an SA-sensor chip. All SPR experiments were then performed, using HBS-EP buffer (10 mM HEPES, 150 mM NaCl, 0.05% *v*/*v* Tween, 3 mM EDTA, pH 7.4).

The SA-sensor chip was prepared based on the manufacturer’s protocol: in brief, biotin sensor was installed to the Nicoya OpenSPR (Nicoya Lifesciences, Kitchener, ON, Canada) sensor holder and 150 μL of 10 mM HCl was injected for surface conditioning at flow rate of 150 μL/min. Then, 150 μL of 0.5 μM SA in HBS-EP running buffer was injected to CH(1 + 2) to coat the biotin sensor at flow rate of 20 μL/min, and 5 min interaction time. A heparin/SA chip was made by immobilizing 150 μL of 50 μg/mL biotinylated heparin in HBS-EP running buffer over flow cell CH_2_ of the SA chip at a flow rate of 20 μL/min. The successful immobilization of heparin was confirmed by the observation of at least 20 resonance unit (RU) increase in the SA-sensor chip.

Competitive solution SPR spectroscopy was conducted by premixing blood (co)-factors IIa (200 nM), Xa (600 nM), AT (200 nM), and HCII (800 nM) with 1 µM each of UFH (positive control), BoSG Fr1, and the three chemical derivatives, desulfated (deS), oversulfated (overS), and alkali-treated (Alk), of BoSG Fr1 in HBS-EP buffer. The mixtures were allowed to equilibrate for 25 min and injected over the heparin surface sensor chip at a flow rate of 20 μL/min, and 5 min injection time. At the end of the sample injection, the running buffer was flowed over the sensor surface to facilitate natural dissociation. After a 10 min dissociation time, the sensor surface was regenerated by injecting it with 150 μL 2 M NaCl to obtain a fully regenerated surface. The response was monitored as a function of time (sensorgram) at 20 °C. The four blood (co)-factors were injected over the heparin/SA-sensor chip, and the binding response (RU) of each co-factor alone was used as a negative control, and UFH as a positive control. All SPR measurements were performed on a Nicoya OpenSPR Rev 4 operated by OpenSPR software (version 4.3.8123.29613) and TraceDrawer analysis software (version 1.9.2).

## 4. Conclusions

Three different sulfated polysaccharides (BoSG Fr1, Fr2 and Fr3) are found in the cell wall of the red alga *B. occidentalis* when the total anionic polysaccharides are subjected to anion-exchange chromatography. Due to the exclusive occurrence of the D enantiomer at the 4-linked *α*-Gal unit in BoSG [17], these polysaccharides are classified as carrageenans. In our previous publication [12], we were able to revisit and revise the structure of the two more sulfated polysaccharides (BoSG Fr2 and Fr3), the two last peaks from the anion-exchange chromatography. All these three polysaccharides clearly exhibit different sulfation contents and chemical groups despite their backbones being composed of both 4-linked *α*- and 3-linked *β*-Gal units. The structure of the first BoSG fraction (Fr1), the first peak from the anion-exchange chromatography, was, for the first time, researched here. Our current results indicate that BoSG Fr1 exhibits large amounts (60% of the total Gal units) of non-sulfated Gal units, along with great amounts of 2-*O*-methylated or 2-*O*-sulfated *α*-AnGal units, and 4-*O*-sulfated or 2,4-di-*O*-sulfated *β*-Gal units. Based on our previous publication [12], the second BoSG (Fr2) shows half of the *α*- and *β*-Gal residues as non-sulfated units, with a lack of *α*-AnGal units, 2-*O*- and 2,3-di-*O*-sulfated *α*-Gal units, and 4-*O*-sulfated *β*-Gal units. The third BoSG (Fr3) is the most sulfated one (2/3 of the total Gal units are sulfated), contains a small quantity of *α*-AnGal units, mostly 2-*O*-sulfated, and the presence of 2-*O*-sulfated or 3-*O*-sulfated *α*-Gal units, and 4-*O*-sulfated or 2,4-di-*O*-sulfated *β*-Gal units. The average MWs of these three BoSG populations are very similar: around 70, 60, and 50 kDa, respectively, for BoSG Fr1, Fr2, and Fr3. In terms of biological activity, BoSG Fr2 has presented overall the best profile among all three BoSG fractions, as noted through SPR-based binding assays, with the key blood co-factors of high affinity for this class of marine polysaccharides: IIa, Xa, AT, and HCII. BoSG Fr2 showed the best molecular interactions with the four blood co-factors investigated in an SPR heparin surface, followed by much weaker interactions of BoSG Fr 1 and the almost total absence of binding properties of BoSG Fr3. This strong biomedical property of BoSG Fr2 might be due to the balance between the presence of a reasonable amount of sulfation (half of the total backbone Gal units) and the lack of the other chemical modifications like the 3,6-anhydro moiety and methylation, as seen in BoSG Fr1 and BoSG Fr3, with their lower binding affinity to the blood-co-factors. In summary, this report, together with our previous work [12], completes both the structural characterization of all three BoSGs and the correlation between their composing structural features and their levels of binding to the four key blood co-factors.

## Figures and Tables

**Table 1:** ^1^H and ^13^C chemical shifts (ppm) ^a^ of the composing units from BoSG Fr1 and from references. Values in bold indicate sulfation sites while values in italic indicate glycosylation sites.

Unit: [Structure] and (Letter Notation)	Source	^1^H1/^13^C1	^1^H2/^13^C2	^1^H3/^13^C3	^1^H4/^13^C4	^1^H5/^13^C5	^1^H6/^13^C6	2-*O*-Me
[→4)-*α*-AnGal-2-(SO_3_^−^)-(1→] (A1)	Fr1	5.39/94.8	**4.27/78.7**	4.41/76.1	*4.16/79.7*	4.03/74.0	3.32, 3.94/64.9	-
[→4)-*α*-AnGal-2(SO_3_^−^)-(1→]	[12]	5.35/94.7	**4.40/76.7**	4.81/78.7	*-*	-	-	-
[→4)-*α*-AnGal-2-*O*-Me-(1→] ^b^ (B1)	Fr1	5.35/96.0	4.09/79.2	4.24/78.9	*3.83/78.5*	3.74/76.1	3.32, 3.94/64.9	3.51/60.1
[→4)-*α*-AnGal-2-*O*-Me-(1→]	[31]	5.31/98.7	3.5578.8	3.85/78.4	*3.65/77.6*	3.4575.3	3.33/69.8	3.51/60.1
[→4)-*α*-AnGal-2(SO_3_^−^)-(1→] (C1)	Fr1	5.32/95.4	**4.41/76.1**	4.81/77.3	*4.16/79.7*	4.05/74.0	3.32, 3.94/64.9	-
[→4)-*α*-AnGal-2(SO_3_^−^)-(1→]	[12]	5.35/94.7	**4.40/76.7**	4.81/78.7	*-*	-	-	-
[→4)-*α*-AnGal-(1→] (D1)	Fr1	5.31/95.6	4.26/70.6	4.87/74.4	*3.83/78.5*	4.29/78.9	3.32, 3.94/64.9	-
[→4)-*α*-AnGal-(1→]	[32]	5.07/94.7	4.08/70.4	4.53/79.7	*4.60/78.5*	4.67/77.1	3.32, 3.94/64.9	-
[→4)-*α*-AnGal-2-*O*-Me-(1→] (E1)	Fr1	5.27/97.0	4.09/78.7	4.22/78.9	*3.83/78.5*	3.74/76.1	3.32, 3.94/64.9	3.51/60.1
[→4)-*α*-AnGal-2-*O*-Me-(1→]	[33]	5.31/98.7	3.5578.8	3.85/78.4	*3.65/77.6*	3.4575.3	3.33/69.8	3.51/60.1
[→4)-*α*-AnGal-2(SO_3_^−^)-(1→] (F1)	Fr1	5.25/95.4	**4.36/76.1**	4.64/74.3	*3.98/78.7*	-	-	-
[→4)-*α*-AnGal-2(SO_3_^−^)-(1→]	[33]	5.27/94.7	**4.66/75.4**	4.78/78.1	*4.72/78.3*	4.70/78.1	4.06. 4.21/70.0	-
[→4)-*α*-AnGal-2(SO_3_^−^)-(1→] (G1)	Fr1	5.16/95.6	**4.29/78.5**	4.41/76.1	*4.13/79.0*	3.96/77.5	3.32, 3.94/64.9	-
[→4)-*α*-AnGal-2(SO_3_^−^)-(1→]	[33]	5.27/94.7	**4.66/75.4**	4.78/78.1	*4.72/78.3*	4.70/78.1	4.06. 4.21/70.0	-
[→4)-*α*-AnGal-(1→] (H1)	Fr1	5.25/92.5	3.79/70.0	4.33/76.2	*4.02/79.2*	4.21/79.0	3.32, 3.94/64.9	-
[→4)-*α*-AnGal-(1→]	[32]	5.07/94.7	4.08/70.4	4.53/79.7	*4.60/78.5*	4.67/77.1	3.32, 3.94/64.9	-
[→4)-*α*-AnGal-(1→] (I1)	Fr1	5.17/92.3	3.69/71.1	3.82/82.3	*4.05/79.2*	3.70/83.6	3.32, 3.94/64.9	-
[→4)-*α*-AnGal-(1→]	[32]	5.07/94.7	4.08/70.4	4.53/79.7	*4.60/78.5*	4.67/77.1	3.32, 3.94/64.9	-
[→3)-*β*-D-Gal-2,4(SO_3_^−^)-(1→] (J1)	Fr1	4.94/101.4	**4.39/76.1**	*4.15/79.7*	**4.97/73.5**	3.95/69.6	3.70/61.1	-
[→3)-*β*-D-Gal-2,4(SO_3_^−^)-(1→]	[12]	4.94/102.4	**4.41/76.7**	*4.18/81.1*	**4.99/74.2**	-	3.77/62.4	-
[→3)-*β*-D-Gal-4(SO_3_^−^)-(1→] (K1)	Fr1	4.80/102.8	3.37/73.2	*4.02/79.2*	**4.88/74.4**	3.82/74.4	3.70/61.1	-
[→3)-*β*-D-Gal-4(SO_3_^−^)-(1→]	[32]	4.70/102.5	3.65/69.4	*4.06/78.2*	**4.91/73.7**	3.87/74.8	3.86/61.2	-
[→3)-*β*-D-Gal-(1→] (L1)	Fr1	4.75/102.0	3.43/73.7	*3.62/84.9*	4.01/70.0	3.38/73.5	-	-
[→3)-*β*-D-Gal-(1→]	[34]	4.79/103.5	3.90/69.9	*3.93/81.9*	4.29/68.3	3.82/74.8	3.87/60.5	
[→3)-*β*-D-Gal-(1→] (M1)	Fr1	4.65/103.7	4.08/73.2	*3.71/83.9*	4.02/70.1	3.38/73.2	3.62/61.1	-
[→3)-*β*-D-Gal-(1→]	[20]	4.57/104.5	3.63/72.1	*3.73/84.1*	4.12/71.4	3.76/73.7	3.77/63.4	-
[→3)-*β*-D-Gal-(1→] (N1)	Fr1	4.58/97.0	3.42/73.7	*3.65/85.0*	4.01/70.1	3.37/73.5	3.61/61.3	-

^a^ The spectra were recorded at 500 MHz in 99.9% D_2_O at 50 °C. Chemical shifts are relative to external trimethylsilylpropionic acid 0 ppm for ^1^H and to methanol for ^13^C. ^b^ Me stands for methyl.

## Data Availability

All data are contained within the manuscript. Data can be provided by the main corresponding author (V.H.P.) upon proper request.

## Supplementary Materials

The following supporting information can be downloaded at: https://www.mdpi.com/article/10.3390/md22020081/s1. Figure S1. Monosaccharide analysis of BoSG Fr1 by LC-MS. The retention time (min) of BoSG Fr1 was compared to a series of monosaccharide standards indicating pure galactose composition.
