# Supplementary material for: Structure and Binding Properties to Blood Co-Factors of the Least Sulfated Galactan Found in the Cell Wall of the Red Alga Botryocladia occidentalis
Source: Mar Drugs. 2024 Feb 9;22(2):81. doi: 10.3390/md22020081 (PMC10890634; doi:10.3390/md22020081)
Supplement: Supplementary file 1 [file marinedrugs-22-00081-s001.zip › marinedrugs-2850575-supplementary.pdf]

SUPPLEMENTARY MATERIAL

Structure and Binding Properties to Blood Co-Factors of the Least Sulfated Galactan Found in the Cell Wall of the Red Alga *Botryocladia occidentalis*

Antim K. Maurya, Hoda A. Ahmed, Anderson DeWitt, Anter A. Shami, Sandeep K. Misra, Vitor H. Pomin

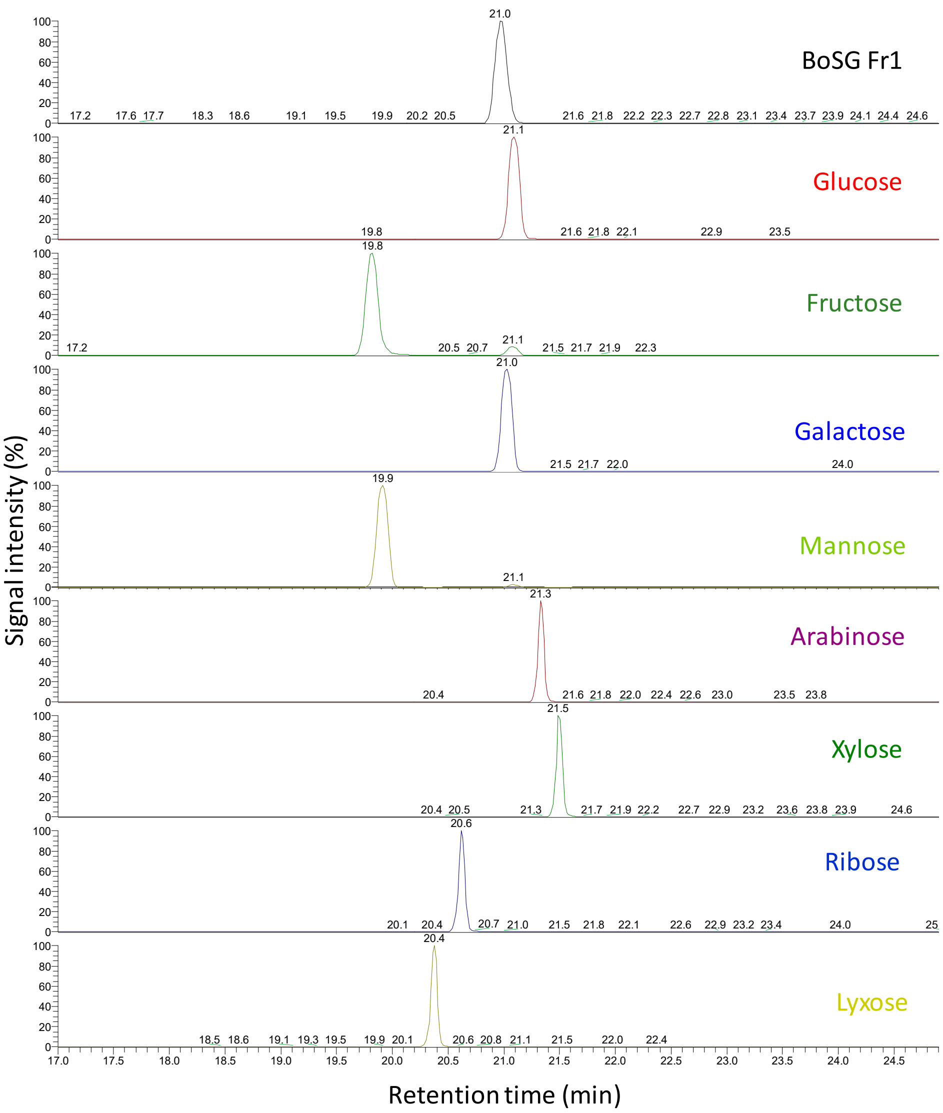

Figure S1. Monosaccharide analysis of BoSG Fr1 by LC-MS. The retention time (min) of BoSG Fr1 was compared to a series of monosaccharide standards indicating pure galactose composition.

Materials and Methods

BoSG Fr1 was dissolved in 0.5 mL of 4 M trifluoroacetic acid in a capped 4 mL ampoule and incubated at 120 ºC for 6 h in an oil bath for hydrolysis. Then, sample was allowed to cool to room temperature, and 50 µL of methanol was added. Acid and solvents were evaporated with a stream of N_2 in a water bath at room temperature. After the addition of 100 µL of methanol, the sample was again evaporated in the ice water bath. Residual methanol was removed by successively evaporation using 2 portions of 100 µL of isopropyl alcohol in the ice water bath. The resulting monosaccharides were dissolved in 100 µL 28 % (v/v) NH_4OH. The monosaccharides obtained from hydrolysis of BoSG Fr1, and the monosaccharide standards Glucose, Fructose, Galactose, Mannose, Arabinose, Xylose, Ribose, and Lyxose, were incubated with 100 µL PMP solution 3-mL ampoule and seal under a N_2 atmosphere. The reaction was allowed to proceed for 40 min at 70 ºC. The samples were cooled to 4 ºC and the reaction was terminated by adding 20 µL 1% glacial acetic acid. The sample was extracted with water and chloroform mixture, centrifuged at 13000 g for 15 min and the supernatant was collected for LC-MS analysis. The samples were then loaded onto a Kinetex C18 Column (2.6 µm, 100Å, 150 x 2.1 mm) using Dionex Ultimate 3000 system (Thermo Fisher Scientific) at the flow rate of 100 µL/min connected to Orbitrap Exploris (Thermo Fisher Scientific). Solvent A was water with 0.1% fluoroacetic acid and the solvent B was acetonitrile with 0.1% fluoroacetic acid. The samples were eluted with the increasing concentration of solvent B and the gradient consisted of 2% B for 3.5 min, increased to 50% B over 20 min, then increased to 95% B over 1 min, held for 2 min, and ramped down to 2% B over 3 min and hold for 8 min. The ion transfer tube temp was set to 4000 V and the full scan range was set at 250-1800 m/z. The top 10 peaks were selected for HCD fragmentation. The percentage of the monosaccharide was calculated based on the area under the curve for the detected monosaccharides.
